# Grazing impact of the calanoid copepods *Acartia* spp. on the toxic dinoflagellate *Alexandrium pseudogonyaulax* in the western coastal waters of Korea

**DOI:** 10.3389/fmicb.2024.1400343

**Published:** 2024-06-19

**Authors:** Moo Joon Lee, Yeong Jong Hwang, Yong Bum Choi, Yeong Du Yoo

**Affiliations:** ^1^Department of Marine Biotechnology, Anyang University, Incheon, Republic of Korea; ^2^School of Earth and Environmental Sciences, College of Natural Sciences, Seoul National University, Seoul, Republic of Korea; ^3^Department of Ocenogaphy, College of Ocean Sciences, Kunsan National University, Kunsan, Republic of Korea

**Keywords:** ingestion, food web, metazooplankton, predator, ecology

## Abstract

Marine dinoflagellate species in the genus *Alexandrium* are well known to produce paralytic shellfish poison as well as common coastal species with cosmopolitan distribution. However, few studies on the feeding of copepods on *Alexandrium* species have been conducted. The toxic dinoflagellate *Alexandrium pseudogonyaulax* contains goniodomin A and causes red tides in many countries. To investigate the relationship between the toxic dinoflagellate *A. pseudogonyaulax* and the calanoid copepods *Acartia* spp., we quantified the ingestion rates of *Acartia* spp. feeding on *A. pseudogonyaulax* as a function of prey concentration. Additionally, we estimated grazing coefficients by integrating data from field observations of *Acartia* spp. and coexisting *A. pseudogonyaulax* with laboratory measurements of ingestion rates obtained during this investigation. Furthermore, we compared the ingestion rates of *Acartia* spp. and other predators feeding on *Alexandrium* species as previously reported. The ingestion rates of *Acartia* spp. on *A. pseudogonyaulax* increased continuously with increasing mean prey concentration. The highest values among the ingestion rate of *Acartia* spp. feeding on *A*. *pseudogonyaulax* was 3,407 cells predator^−1^ d^−1^ (4,872 ng C predator^−1^ d^−1^) at the given prey concentration. The calculated grazing coefficients for *Acartia* spp. on *A. pseudogonyaulax* in Shiwha Bay, Korea, were up to 0.073 d^−1^. The results of this study suggest that *A. pseudogonyaulax* may decrease or maintain the population of *Acartia* spp. in marine food webs.

## 1 Introduction

Marine dinoflagellates and copepods are important components of aquatic environments (Calbet et al., 2003; Jeong et al., 2010, 2021; Turner et al., 2012; Kim et al., 2013). Marine dinoflagellates are ubiquitous species that can dominate the biomass and density of the marine environment (Jeong et al., 2010, 2013, 2021; Hansen, 2011; Nagarkar et al., 2018; Goswami et al., 2020; Telesh et al., 2021). Copepods are major zooplankton in marine food webs and are effective grazers of protist prey species and sometimes control dinoflagellate populations (Watras et al., 1985; Campbell et al., 2005; Jeong et al., 2010; Kim et al., 2013). Therefore, to understand the roles and population dynamics of dinoflagellates in marine communities, growth and mortality due to zooplankton predation must be explored.

Marine dinoflagellate species of the genus *Alexandrium* are widely distributed and sometimes cause red tides or harmful algal blooms (Anderson, 1997; Cembella et al., 2000; Grattan et al., 2016; Kremp et al., 2019; Shin et al., 2021). Several species of *Alexandrium* have been well–studied for their physiological and ecological properties, such as toxin profiles, growth rates, distributions, and predation, because they often cause large-scale shellfish mortality and human illnesses due to the toxins they produce (Parkhill and Cembella, 1999; Cembella et al., 2000, 2002; Navarro et al., 2006; Etheridge, 2010; Bill et al., 2016; Grattan et al., 2016; Kim et al., 2016; Kang et al., 2018). Many *Alexandrium* species produce toxins, such as paralytic shellfish poisoning (PSP) and other allelochemicals, which are potentially transferred to marine organisms in higher trophic levels (Cembella et al., 2000; Turner et al., 2005; Sephton et al., 2007; Ma et al., 2011; Anderson et al., 2012; Tillmann et al., 2016). Therefore, they are of interest to government officials, fish consumers, and marine scientists (Alcala et al., 1988; Anderson et al., 2012). Thus, understanding the interactions between dinoflagellates and their consumers is important for understanding the diversity of red tides and harmful algal species (Turner et al., 2006; Jeong et al., 2010; Kim et al., 2013; Yoo et al., 2013; Kang et al., 2018).

In this study, we isolated and established a clonal culture of *Alexandrium pseudogonyaulax* from the coastal waters of Korea (Yoo et al., 2023). In many countries, this species produces goniodomin A, which cause red tides (Matsuoka and Fukuyo, 2003; Bravo et al., 2006; Kremp et al., 2019). Previously, *A. pseudogonyaulax* has been shown to be a phototrophic dinoflagellate. However, this species is a mixotrophic dinoflagellate (Blossom et al., 2012). Several studies have been performed on the taxonomy, ecology, physiology, distribution, bioinformatics, and cysts of this species (Montresor and Marino, 1996; Matsuoka and Fukuyo, 2003; Blossom et al., 2012; Triki et al., 2016; Yoo et al., 2023). However, few studies have been conducted on the mortality of *A. pseudogonyaulax* caused by grazers. Grazing can play an important role in dinoflagellate population dynamics (Watras et al., 1985; Turner et al., 2006; Jeong et al., 2010; Yoo et al., 2013). Copepods are effective grazers of several dinoflagellates (Watras et al., 1985; Jeong et al., 2010; Kim et al., 2013). Thus, to understand the roles and population dynamics of *A. pseudogonyaulax*, the predator–prey relationships between *A. pseudogonyaulax* and copepods was investigated. Additionally, we compared the ingestion rates of *Acartia* spp. in the present study with those of other *Alexandrium* species and dinoflagellates reported in the literature. The results of this study provide a basis for understanding the interactions between *A. pseudogonyaulax* and *Acartia* spp. and their population dynamics in marine planktonic food webs.

## 2 Materials and methods

### 2.1 Preparation of experimental organisms

For isolation and culture of *Alexandrium pseudogonyaulax,* plankton samples collected with Niskin sampler were taken from Shiwha Bay, Korea when the water temperature and salinity were 25.4°C and 23.9, respectively (Table 1). These samples were screened through a 202–Nitex mesh and placed in 6–well tissue culture plates (SPL lifesciences, Gyeonggido, Korea). A clonal culture of *A. pseudogonyaulax* was established by performing two serial single–cell isolations. As the concentration of *A. pseudogonyaulax* increased, this species was subsequently transferred to 50–mL and 500–mL polycarbonate (PC) bottles containing fresh f/2–Si medium (Guillard and Ryther, 1962). Freshly filtered seawater was used to fill bottles containing the f/2-Si medium and *A. pseudogonyaulax*. The capped bottles were then incubated at 20°C under illumination of 20 μmol photons m^−2^ s^−1^ of cool white fluorescent light on a 14:10 h light:dark cycle. Once dense cultures of *A. pseudogonyaulax* were obtained, the cells were transferred to new 2–L PC bottles containing fresh f/2-Si medium approximately 3 weeks before the feeding experiments were conducted at a temperature of 15°C.

**Table 1:** Isolation and maintenance conditions of the experimental organisms.

Organisms	Location	Temp (°C)	Sal	ESD (μm)	Prey species	Concentration (cells mL^−1^)
*Alexandrium pseudogonyaulax*	Shiwha Bay, Korea	25.4	23.9	24.8		
*Acartia* spp. (*A. hongi* and *A. omorii*)	Shiwha Bay, Korea	7.3	27.2		*Prorocentrum cordatum*	12,000

ESD, equivalent spherical diameter.

Copepods were collected Shiwha Bay, Korea, using a 303 μm mesh net when water temperature and salinity were 7.3°C and 27.2, respectively (Table 1). The copepods were acclimatized in a 15°C room in the presence of *Prorocentrum cordatum* for 10 days. Adult female *Acartia* spp. (*A. hongi* and *A. omorii*) were used in the experiments. *A. hongi* and *A. omorii* which co-occur in the western coastal waters of Korea, are very similar and it is impossible to distinguish between these two species when they are alive (Soh and Suh, 2000).

The mean equivalent spherical diameter (ESD) of live *A. pseudogonyaulax* was measured using an electron–particle counter (Coulter Multisizer II; Coulter Corporation, Miami, Florida, United States). The carbon content of this species was estimated based on the cell volume according to Menden-Deuer and Lessard (2000).

### 2.2 Swimming speed

A dense culture (*ca.* 1,500 cells mL^−1^) of *A. pseudogonyaulax,* which grew photosynthetically under a 14:10 h light:dark cycle at 20 μmol photons m^−2^ s^−1^ in f/2–Si medium was transferred to a 250–mL PC bottle. Subsequently, an aliquot from the bottle was transferred to a 50–mL cell culture flask and allowed to acclimate for 30 min. The observations were conducted at 20°C using a video analyzing system (SV-C660, Samsung) and a CCD camera (KP-D20BU, Hitachi). The video camera was focused on a field of view within the cell culture flask and observed as a single field under a dissecting microscope at 50× magnification. The mean and maximum swimming velocities of all *A. pseudogonyaulax* cells in motion within the first 10 min were recorded and analyzed. The linear displacement of the cells within a single–frame playback was measured to calculate the average swimming speed. The swimming velocities of 30 cells were assessed.

### 2.3 Ingestion rates of *Acartia* spp. on *Alexandrium pseudogonyaulax*

This experiment was designed to measure the ingestion and clearance rates of *Acartia* spp. on *A. pseudogonyaulax* as a function of prey concentration (Table 2). Adult female *Acartia* spp. (a combination of *A. hongi* and *A. omorii*) were used in the present study.

**Table 2:** Experimental design for feeding by the copepods *Acartia* spp. on *Alexandrium pseudogonyaula*x.

Abundance of *Alexandrium pseudogonyaulax*	Abundance of *Acartia* spp. (*A. hongi* and *A. omorii*)
0	20
25 (36)	20
63 (91)	20
132 (189)	20
265 (379)	20
688 (983)	20
1,429 (2,044)	20

The numbers are the initial abundances (cells mL^−1^ for *A. pseudogonyaulax* and inds. L^−1^ for *Acartia* spp.) of *A. pseudogonyaulax* and *Acartia* spp. Values in the parentheses in *A. pseudogonyaulax* are the abundances in ng C mL^−1^.

For the feeding experiment, the initial concentrations of *A. pseudogonyaulax* were dertermined using an autopipette to deliver predetermined volumes of known cell concentrations to the bottles and those of *Acartia* spp. were obtained by individually transferring *Acartia* spp. using a pasteur pipette. Triplicate 500–mL PC bottles (mixtures of predator and prey) and triplicate control bottles (*A. pseudogonyaulax* prey only) were set up for each predator–prey combination. In order to maintain consistent water conditions, the water from the predator culture was passed through a 0.7 μm GF/F filter before being added to the prey control bottles. The volume of the filtered predator culture added to the experimental bottles for each predator–prey combination was matched with an equal amount of filtered water added to the prey control bottles. All the bottles were filled to capacity with freshly filtered seawater and capped. To determine the initial predator and prey densities, a 10–mL sample was extracted from each bottle and fixed with 1% Lugol’s solution for fixation. The fixed sample were examined using a light microscope to determine the abundance of predator and prey species. The cells in three 1–mL Sedgwick-Rafter chambers (SRCs) were counted to determine the actual densities of predator and prey species. The bottles were refilled to capacity with freshly filtered seawater, capped, and placed on rotating wheels at a temperature of 15°C, following the conditions outlined earlier. We considered any dilution of the cultures associated with refilling the bottles when determining the clearance rate. A 10–mL aliquot was taken from each bottle after 24 and 48–h incubation periods and fixed with 1% Lugol’s solution. The abundance of prey species was determined by counting all or > 200 cells in three 1–mL SRCs. Following sub–sampling, the bottles were filled with freshly filtered seawater and placed back on rotating wheels. After incubation for 48 h, the *Acartia* spp. were counted. The mortality of *Acartia* spp. occurred until the end of the incubation period. The ingestion and clearance rates were calculated using the equations of Frost (1972).

### 2.4 Grazing impact

We calculated the grazing coefficients attributable to *Acartia* spp. on *A. pseudogonyaulax* by combining field data on the abundances of *Acartia* spp. and *A. pseudogonyaulax* with the ingestion rates of *Acartia* spp. on *A. pseudogonyaulax* obtained in the present study. Data on the abundance of *Acartia* spp. and co-occurring *A. pseudogonyaulax* used in this estimation were obtained from water samples from Shiwha Bay, Korea using real-time PCR for *A. pseudogonyaulax* and cell counting for *Acartia* spp.

The grazing coefficients (g, d^−1^) were calculated as follows:


(1) g=CR×GC×24

where CR is the clearance rate (mL predator^−1^ h^−1^) of a *Acartia* spp. on *A. pseudogonyaulax* at a given prey concentration and GC is the predator concentration (cells mL^−1^). The CR values were calculated as follows:


(2) CR=IR/X

where IR is the ingestion rate (cells predator^−1^ h^−1^) of the predator on the prey and *X* is the prey concentration (cells mL^−1^).

### 2.5 Species-specific primer and probe design and specificity analysis

We developed species-specific primer and probe set for *A. pseudogonyaulax* and obtained the sequences of the internal transcribed spacer region of ribosomal DNA (ITS rDNA) of *A. pseudogonyaulax* and other dinoflagellate species belonging to the *Alexandrium* genus and related dinoflagellate species from GenBank. These sequences were aligned using MEGA v.11. *A. pseudogonyaulax* specific primers and probe were developed by searching the arrangement for unique portions of the ITS rDNA sequences for *A. pseudogonyaulax*. Primer and probe sequences were analyzed using Primer 4 (Whitehead Institute for Biomedical Research, Cambridge, MA, United States) and Oligo Calc: Oligonucleotide Properties Calculator (Kibbe, 2007) to investigate the optimal melting temperature and secondary structure, respectively. Primers and probe were synthesized by Bioneer (Table 3). The probe was dual labeled with the fluorescent dyes FAM and BHQ1 at the 5′ and 3′ ends.

**Table 3:** Sequences of the primers and probe for *Alexandrium pseudogonyaulax* used in this study.

Target gene	Analysis	Primer name		Primer sequence (5′ – 3 ′)	References
ITS rDNA	PCR	ITSF2	Forward	TACGTCCCTGCCCTTTGTAC	Litaker et al. (2003)
		LSU500R	Reverse	CCCTCATGGTACTTGTTTGC	Litaker et al. (2003)
	qPCR	Apsudo_F	Forward	GAAGGTGTGCTTGATCCAATGTAA	This study
		Apseudo_R	Reverse	CACACACAATGGCAAACCTTTCAC	This study
		Apseudo_P	Probe	TGCTTATGGGCTTCTG	This study

ITS, Internal transcribed spacer; PCR, polymerase chain reaction; qPCR, quantitative real-time PCR.

Specificity analysis of the primer and probe sets for *A. pseudogonyaulax* was performed using DNA extracts of *A. pseudogonyaulax* and related dinoflagellate species in the Family Pyrocystaceae. The qPCR reaction mixture contained 1 μL of DNA template, 0.2 μM of specific forward and reverse primers, 0.15 μM of the specific probe, 5 μL of qPCRBIO Probe Separate-ROX (Genepole, Gwangmyeong, Korea), and deionized sterilized water (DDW; Bioneer), with a final total volume of 10 μL. The qPCR assay was conducted using the Rotor-Gene Q (Qiagen, Hilden, Germany). The cycling conditions were initialized with a denaturation step at 95°C for 3 min, followed by 40 cycles of 10 s at 95°C for 10 s, and 58°C for 40 s.

### 2.6 Standard curve construction

A standard curve for exploring the abundance of *A. pseudogonyaulax* was constructed using a qPCR. DNA was extracted from the culture of *A. pseudogonyaulax* (4,200 cells mL^−1^) in the growth phase using the AccuPrep Genomic DNA Extraction Kit (Bioneer), targeting 1, 10, 100, 1,000, 2,000, and 4,000 *A. pseudogonyaulax* cells. The qPCR assay was performed using the reaction mentioned above under the following thermal cycling conditions: 95°C for 3 min, followed by 45 cycles of 10 s at 95°C for 10 s, and 58°C for 40 s.

### 2.7 Quantification using qPCR

We developed species-specific primer and probe set for *A. pseudogonyaulax*, and obtained the sequences of the internal transcribed spacer region of ribosomal DNA (ITS rDNA) of *A. pseudogonyaulax* and related dinoflagellates.

The previously mentioned qPCR assay conditions were used to analyse the abundance of *A. pseudogonyaulax* in field samples. The DNA from each sample was amplified four times to ensure the accuracy of results. The sample using DDW as the template was used as a negative control, whereas the one used to construct a standard curve was used as positive and standard control.

## 3 Results

### 3.1 Ingestion rates of *Acartia* spp. on *Alexandrium pseudogonyaulax*

**Figure 1:**
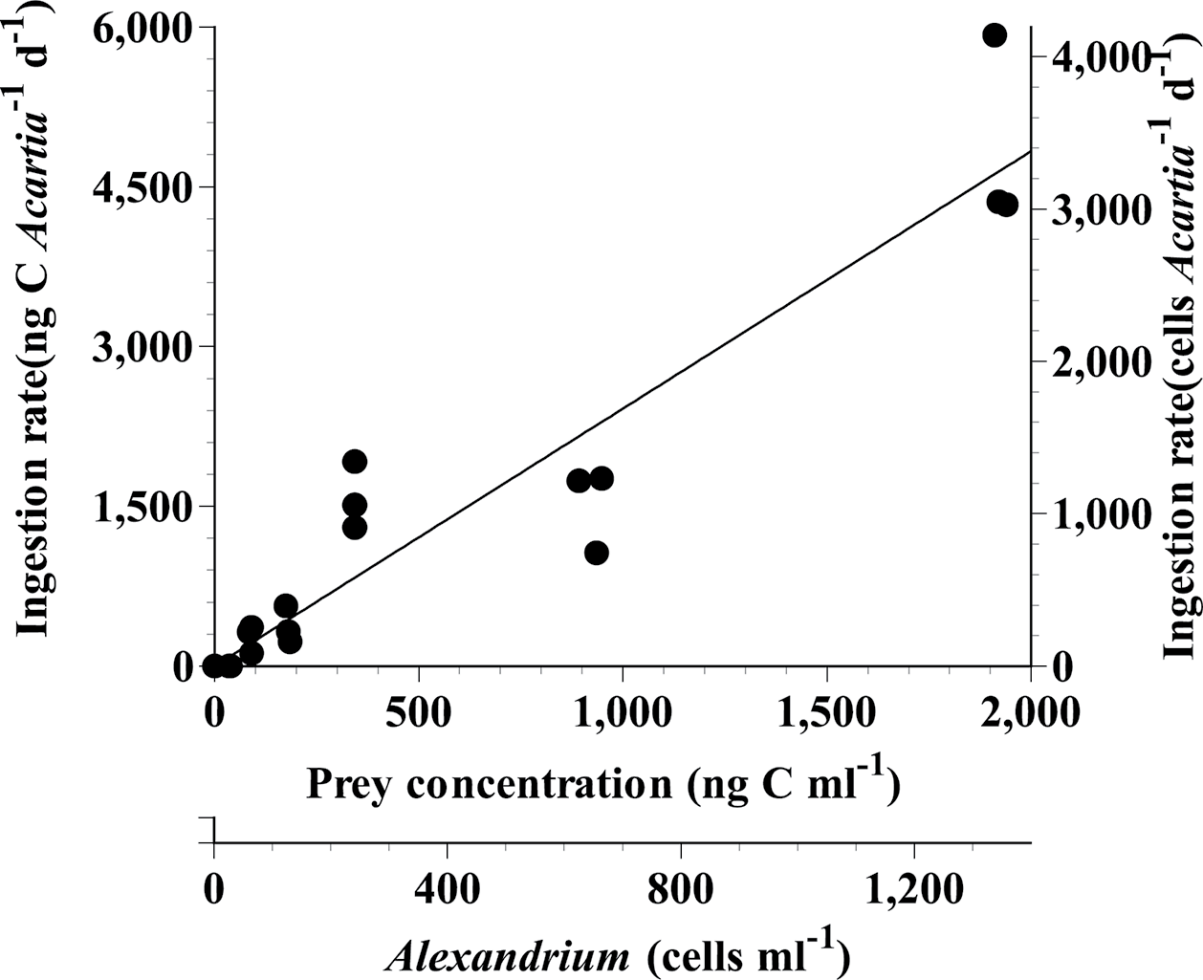
Ingestion rates (IR) of *Acartia* spp. (*A. hongi* and *A. omorii*) feeding on *Alexandrium pseudogonyaulax* as a function of mean prey concentration (x). Symbols represent treatment means ± 1 SE. The curves were fitted using the linear regression equation. IR (ng C *Acartia*^−1^ d^−1^) = 2.42 × (x), *r*^2^ = 0.938.

The ingestion rate of *Acartia* spp. on *A. pseudogonyaulax* continuously increased with increasing prey concentration (Figure 1). The highest ingestion and clearance rates of *Acartia* spp. on *A. pseudogonyaulax* at the given prey concentration was 3,407 cells *Acartia*^−1^ d^−1^ (4,872 ng C *Acartia*^−1^ d^−1^) and 192 mL *Acartia*^−1^ h^−1^, respectively.

### 3.2 Grazing impact

**Figure 2:**
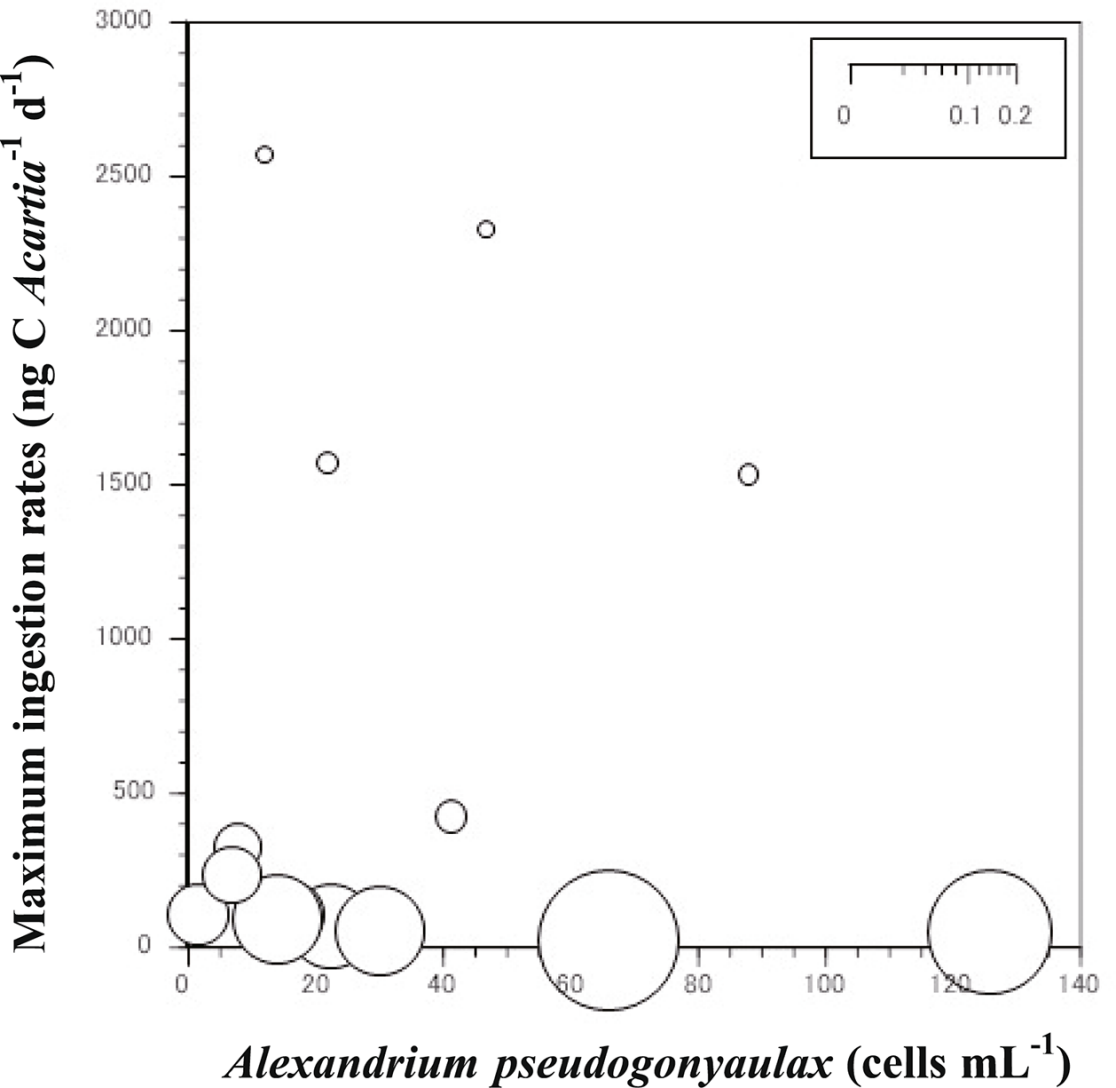
Calculated grazing coefficients (g, d^−1^) of the *Acartia* spp. (*A. hongi* and *A. omorii*) in relation to the population of co-occurring *Alexandrium pseudogonyaulax* in the western coastal waters of Korea.

The grazing coefficients attributable to *Acartia* spp. on co-occurring *A. pseudogonyaulax* in Shiwha Bay, Korea were affected by the abundance of *Acartia* predators (Figure 2). The abundance of *Acartia* spp. and *A. pseudogonyaulax* were 1.5–126.0 cells mL^−1^ and 2–2,570 ind. m^−3^, respectively. The grazing coefficients attributable to *Acartia* spp. on co-occurring *A*. *pseudogonyaulax* were 0.001 to 0.073 d^−1^ (i.e., up to 7% of *A. pseudogonyaulax* population could be removed by the copepod *Acartia* spp. in a day).

## 4 Discussion

**Figure 3:**
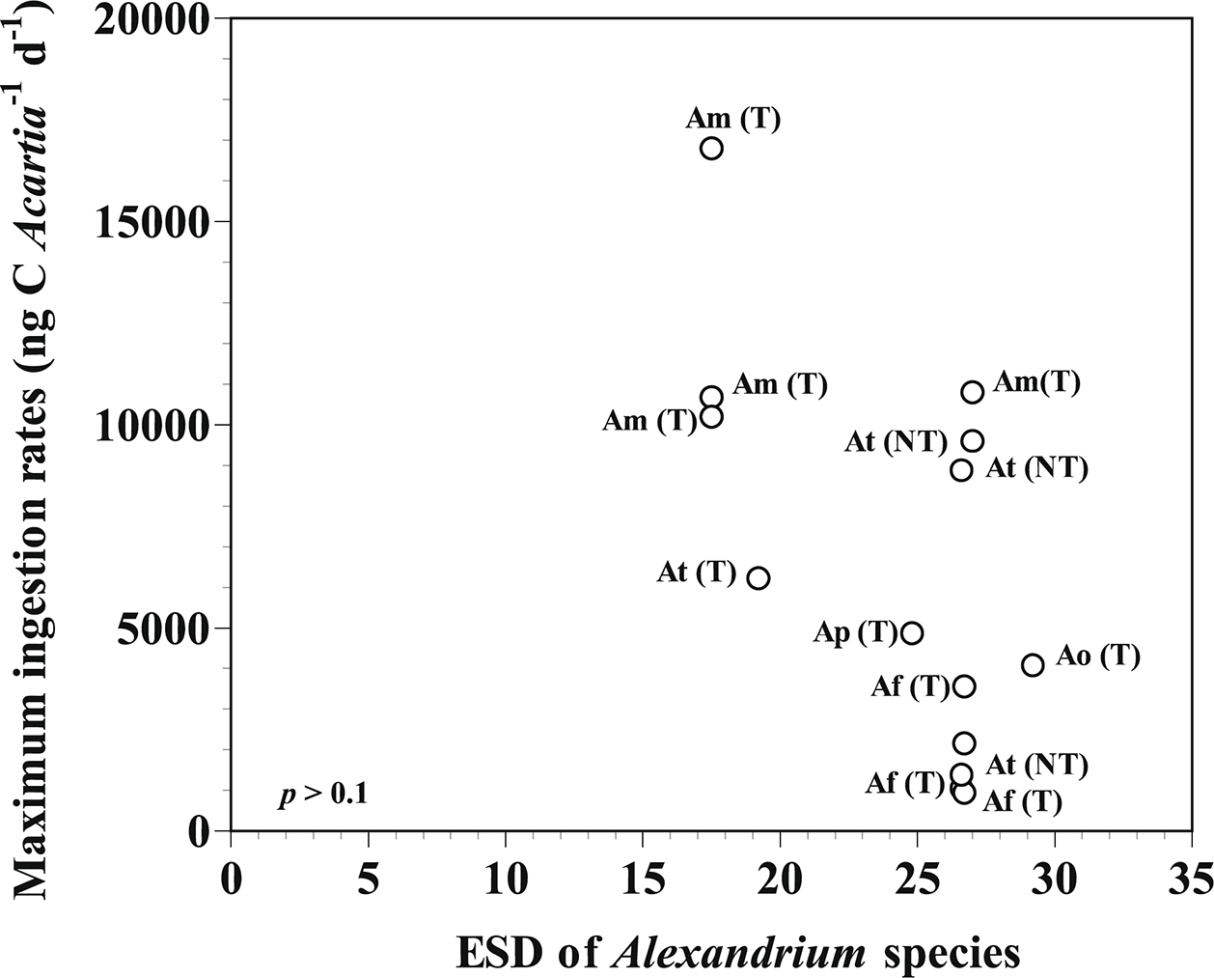
Maximum ingestion rates of *Acartia* spp. feeding on *Alexandrium* prey species as a function of prey size (ESD: equivalent spherical diameter, μm). The *p*-value was *p* > 0.1 (linear regression ANOVA). Af (T): toxic strain of *A. fundyense*; Am (T): toxic strain of *A. minutum*; Ao (T): toxic strain of *A. ostenfeldii*; Ap (T): toxic strain of *A. pseudogonyaulax*; At (T): toxic strain of *A. tamarense*; At (NT): non-toxic strain of *A. tamarense.* The data were obtained from Calbet et al. (2003), Colin and Dam (2002, 2003, 2007), da Costa and Fernández (2002), da Costa et al. (2008), Sopanen et al. (2011), Teegarden (1999), and Teegarden et al. (2003, 2008).

The calanoid copepod *Acartia* is a major components metazooplankton in marine environments (Kim et al., 2013; Rice et al., 2015; Lee et al., 2017). Several *Acartia* species such as *Acartia bifilosa*, *Acartia grani*, *Acartia hudsonica,* and *Acartia tonsa* feed on *Alexandrium* spp., including toxic strains of *Alexandrium fundyense, Alexandrium minutum, Aleandrium ostenfeldii,* and *Alexandrium tamarense* and non-toxic strain of *A. tamarense* (Teegarden, 1999; Colin and Dam, 2002, 2003, 2007; da Costa and Fernández, 2002; Teegarden et al., 2003, 2008; da Costa et al., 2008; Sopanen et al., 2011). Among the maximum ingestion rates (MIRs) of *Acartia* grazers on *Alexandrium* prey species, the MIRs were not significantly correlated with the prey (19–28 μm of equivalent spherical diameter) and predator sizes (Figure 3). *Acartia* spp. (*A. hongi* and *A. omorii*) were significantly larger than *A. grani* and *A. tonsa* (Table 4).

**Table 4:** Comparison of maximum ingestion rates of *Acartia* species on *Alexandrium* species.

Prey species	Temp	Predator species	MIR	References
*Alexandrium pseudogonyaulax* (T)	20.0	*Acartia* spp. (*A. hongi* and *A. omorii*)	4,872	This study
*Alexandrium fundyense* (T)	12.0	*Acartia hudsonica*	10,800	Teegarden et al. (2008)
*Alexandrium fundyense* (T)	14.0	*Acartia hudsonica*	1,060	Colin and Dam (2002)
*Alexandrium fundyense* (T)	14.0	*Acartia hudsonica*	3,563	Colin and Dam (2003)
*Alexandrium fundyense* (T)	17.0	*Acartia hudsonica*	948	Teegarden et al. (2003)
*Alexandrium fundyense* (T)	19.0	*Acartia tonsa*	2,160	Teegarden (1999)
*Alexandrium mimutum* (T)	15.5	*Acartia grani*	10,680	Calbet et al. (2003)
*Alexandrium mimutum* (T)	17.5	*Acartia grani*	10,200	da Costa and Fernández (2002)
*Alexandrium mimutum* (T)	17.5	*Acartia grani*	16,800	da Costa et al. (2008)
*Alexandrium osfeldii* (T)	11.0	*Acartia bifilosa*	4,080	Sopanen et al. (2011)
*Alexandrium tamarense* (T)	14.0	*Acartia hudsonica*	6,220	Colin and Dam (2007)
*Alexandrium tamarense* (NT)	14.0	*Acartia hudsonica*	1,390	Colin and Dam (2002)
*Alexandrium tamarense* (NT)	14.0	*Acartia hudsonica*	9,600	Teegarden et al. (2008)
*Alexandrium tamarense* (NT)	19.0	*Acartia tonsa*	8,880	Teegarden (1999)

ESD, equivalent spherical diameter (μm); Temp, temperature (°C); MIR, maximum ingestion rate (ng C Acartia^−1^ d^−1^); T, toxic strain; NT, non-toxic strain.

Among the MIRs of *Acartia* grazers on *Alexandrium* prey species, the MIR of *A. hudosonica* on the toxic of *A. tamarense* strain was higher than that on the non-toxic strain of *A. tamarense* (Colin and Dam, 2007; Teegarden et al., 2008). Additionally, the MIR of *A. tonsa* on the toxic of *A. fundyense* strain was lower than that on the non-toxic strain of *A. tamarense* (Teegarden, 1999). Thus, the toxicity of *Alexandrium* prey species probably did not affect the MIRs of *Acartia* grazers.

Among the MIRs of *Acartia* grazers on *Alexandrium* prey species, the MIR of *Acartia* spp. on *A. pseudogonyaulax* was higher than *A. bifilosa* on *A. ostenfeldii*, but lower than that of *A. grani* on *A. minutum* (da Costa and Fernández, 2002; da Costa et al., 2008). Many *Alexandrium* species contain PSP toxins; however, the subgenus *Gessnerium* does not produce PSP toxins (Balech, 1995). *A. pseudogonyaulax* belonging to the subgenus *Gessnerium* may not produce PSP toxins, but may produce goniodomin A (Balech, 1995; Matsuoka and Fukuyo, 2003; Bravo et al., 2006). Furthermore, *A. pseudogonyaulax* is a mixotrophic species when mucus traps are used to immobilize prey cells prior to ingestion (Blossom et al., 2012). Therefore, the mucus trap excreted by *A. pseudogonyaulax* may not only function to effectively accumulate toxins but also be used to avoid encounters and ingestion by potential predators.

The motility of dinoflagellates is not only relevant to potential predators but is also important for resource availability (Buskey, 1997; Tillmann and Reckermann, 2002; Jeong et al., 2015, 2017). The swimming speed of *A. pseudogonyaulax* (*n* = 30) was 263–512 μm s^−1^. The average (±standard error) swimming speed of *A. pseudogonyaulax* was 372 (±12) μm s^−1^. The maximum swimming speed of *A. pseudogonyaulax* was faster than that of *A. minutum* but slower than that of *A. tamarense* (Kang et al., 2018). Thus, the swimming speed of *Alexandrium* prey species probably did not affect the MIRs of *Acartia* grazers. Other properties, such as the biochemical factors of *Alexandrium* species, may affect the ingestion of *Acartia* grazers more than their physical and behavior properties.

Grazing impacts calculated by using field observation data of *Acartia* spp. and coexisting *A. pseudogonyaulax* with laboratory measurements of ingestion rates suggest that up to 7% of *A. pseudogonyaulax* populations may be eliminated in a day by the copepods *Acartia* spp. Therefore, the copepod *Acartia* species could have a considerable potential grazing impact on *Alexandrium* populations in Shiwha Bay.

Few studies have been conducted on the grazing effects of copepods on *Alexandrium* species in the field. The grazing effect of *A. hudsonica* on *Alexandrium* spp. was 0.8 d^−1^ at Cundy’s Harbor (Campbell et al., 2005). In Cape Cod embayment, the grazing pressure of *A. hudsonica* feeding on *A. tamarense* was less than 1% (Turner and Anderson, 1983). Additionally, the grazing coefficients of the copepods such as *Acartia granii* and *Oithona davisae* on *A. minutum* were 0.00003–0.00007 d^−1^ (i.e., up to 0.007% of *Alexandrium* populations could be removed by the copepod populations in a day) in the Arenys de Mar harbor (Calbet et al., 2003). Thus, the copepod *Acartia* species sometimes have a considerable potential grazing impact on populations of *Alexandrium.*

## 5 Conclusion

The present study investigated the grazing by calanoid copepods *Acartia* spp. on the toxic dinoflagellate *Alexandrium pseudogonyaulax*. The grazing of *Acartia* spp. can affect the abundance of *Alexandrium* populations in many countries. A total of 34 *Alexandrium* species have been reported, but there have been few studies on grazing by metazooplankton on *Alexandrium* spp. (Calbet et al., 2003; Campbell et al., 2005; Guiry and Guiry, 2020). Therefore, constant investigation of feeding by dominant copepods on *Alexandrium* species would be worthwhile to enhance our understanding of the interactions and population dynamics between the copepods and dinoflagellates in natural marine ecosystems.

## Data availability statement

The datasets presented in this study can be found in online repositories. The names of the repository/repositories and accession number(s) can be found in the article/supplementary material.

## Author contributions

ML: Conceptualization, Investigation, Writing – original draft. YH: Investigation, Writing – original draft. YC: Conceptualization, Investigation, Writing – review & editing. YY: Conceptualization, Investigation, Writing – original draft, Writing – review & editing.
